# Improving Degradation of Polycyclic Aromatic Hydrocarbons by *Bacillus atrophaeus* Laccase Fused with *Vitreoscilla* Hemoglobin and a Novel Strong Promoter Replacement

**DOI:** 10.3390/biology11081129

**Published:** 2022-07-27

**Authors:** Luyao Wang, Yuzhi Tan, Shengwei Sun, Liangjie Zhou, Guojun Wu, Yuting Shao, Mengxi Wang, Zhihong Xin

**Affiliations:** Key Laboratory of Food Processing and Quality Control, College of Food Science and Technology, Nanjing Agricultural University, Nanjing 210095, China; 2019108051@njau.edu.cn (L.W.); 2019108052@njau.edu.cn (Y.T.); 2019208021@njau.edu.cn (S.S.); 2020108050@stu.njau.edu.cn (L.Z.); 2020108049@stu.njau.edu.cn (G.W.); 2018208019@njau.edu.cn (Y.S.); 2020208023@stu.njau.edu.cn (M.W.)

**Keywords:** thermostable laccase, PAH biodegradation, fusion expression, promoter screening, strong promoter replacement

## Abstract

**Simple Summary:**

Polycyclic aromatic hydrocarbons (PAHs) are a large group of persistent organic pollutant chemicals that have significant carcinogenic effects on humans and animals. Degradation of PAHs by microbial laccase is a low-cost and high-efficiency pipeline, but the catalytic activity and expression level for most of the native bacterial laccases are low, limiting their wide use in industry. A recombinant enzyme called LacH5 showed high-efficiency PAH degradation activity, and its degradation activity was further improved by fusion expression and the expression level was augmented by a novel strong promoter replacement. The results from the current investigation provide new insights and strategies for PAH degradation and the development of a new candidate laccase for PAH biodegradation.

**Abstract:**

Laccases catalyze a variety of electron-rich substrates by reducing O_2_ to H_2_O, with O_2_ playing a vital role as the final electron acceptor in the reaction process. In the present study, a laccase gene, *lach5*, was identified from *Bacillus atrophaeus* through sequence-based screening. LacH5 was engineered for modification by fusion expression and promoter replacement. Results showed that the purified enzyme LacH5 exhibited strong oxidative activity towards 2,2’-azinobis(3-ehtylbenzothiazolin-6-sulfnic acid) ammonium salt (ABTS) under optimum pH and temperature conditions (pH 5.0, 60 °C) and displayed remarkable thermostability. The activity of the two fusion enzymes was enhanced significantly from 14.2 U/mg (LacH5) to 22.5 U/mg (LacH5-vgb) and 18.6 U/mg (Vgb-lacH5) toward ABTS after LacH5 fusing with *Vitreoscilla* hemoglobin (VHb). Three of six tested polycyclic aromatic hydrocarbons (PAHs) were significantly oxidized by two fusion laccases as compared with LacH5. More importantly, the expression level of LacH5 and fusion protein LacH5-vgb was augmented by 3.7-fold and 7.0-fold, respectively, by using a novel strong promoter replacement. The results from the current investigation provide new insights and strategies for improving the activity and expression level of bacterial laccases, and these strategies can be extended to other laccases and multicopper oxidases.

## 1. Introduction

Laccase (EC1.10.3.2) is a multicopper oxidase that coordinates four copper ions and oxidizes a wide range of phenolic and nonphenolic aromatic substrates while reducing O_2_ to H_2_O simultaneously [1]. Currently, laccases have been found in plants, fungi, and bacteria. Most research focuses on fungal laccases; however, bacterial laccases exhibit the most significant advantages of high thermostability and activity over a wide pH range [2]. Thus, bacterial laccases are regarded as promising green biological tools and suitable for industrial applications. An increasing number of bacterial laccase genes have been well studied and characterized, including *Bacillus* genera-derived CotA-laccases from *B. subtilis* [3,4], *B. pumilus* [5], *B. licheniformis* [6] *B. amyloliquefaciens* [7], *B. sphaericus* [8], *B. altitudinis* [9], *B. safensis* [10], and *B. cereus* [11]. Furthermore, *Bacillus* laccase has been reported to be a potential biocatalyst for various industrial applications, like pulp biobleaching [12], degrading organ-phosphorous pesticides [13], degrading aromatic organic pollutants [14], etc.

The structures of bacterial laccases have revealed the presence of three bound types of copper ions, according to magnetic and spectroscopic properties, which are named type I (T1), type II (T2), and double type III (T3) copper ions [5,15,16]. The primary reaction mechanism of laccase involves the formation of two H_2_O molecules with the concomitant oxygen molecule gaining electrons [2]. O_2_ is reduced to H_2_O in the trinuclear copper center constituted by type II and type III copper ions [16], and the quantity of dissolved oxygen available plays a pivotal role in the reaction.

Polycyclic aromatic hydrocarbons (PAHs) are a large group of persistent organic pollutant chemicals with two or more fused aromatic rings [17]. PAHs are widespread environmental contaminants formed by incomplete combustion of organic materials such as coal, oil, and natural gas [18]. The PAHs entering the atmosphere can be transported long distances before being deposited into soil, vegetation, or water by atmospheric precipitation [19]. Of all PAHs, 16 have been listed as priority pollutants by the US Environmental Protection Agency (EPA) [20]. Several of the PAHs, such as benz[a]anthracene (BaA), benzo[b]fluoranthene (BbF), and benzo[a]pyrene (BaP), have caused organ damage and cancer in animal laboratory studies [21,22,23]. The carcinogenicity of PAHs also increases with increasing molecular weight [24], like BaP with five aromatic rings, which is considered one of the most carcinogenic PAHs [25]. The increased awareness of the various adverse effects on the ecosystem and human health has led to a dramatic increase in the search for safe and eco-friendly approaches for reducing and removing PAHs. Among the known solutions, biodegradation offers a safe, low-cost, and high-efficiency pipeline.

Compared with that of fungal laccases, the industrial application of bacterial laccases still faces many obstacles, primarily because of low expression levels and catalytic activity and the high cost of mediators, which limits native laccase application in various industrial processes [26]. Several strategies have been applied to improve these properties, including the addition of inducers, physicochemical parameter optimization, chemical modification, and genetic engineering [27]. However, most of these approaches are complicated, laborious, and expensive for industrial production, and only a few bacterial laccases with high activity and high yield are available. Therefore, further research should focus on discovering laccases from microorganisms and improving their expression level and catalytic efficiency.

In the present study, laccase gene *lach5* was obtained by sequence-based screening from *B. atrophaeus*. The corresponding laccase gene was cloned and overexpressed in *E. coli* BL21 (DE3). The recombinant enzyme was purified and biochemically characterized. The activity of LacH5 was enhanced remarkably by fusion expression with *Vitreoscilla* hemoglobin, and the expression level was improved significantly by a new strong promoter replacement. The degradation activity of the recombinant enzymes was tested on six PAHs.

## 2. Materials and Methods

### 2.1. Bacterial Strains, Plasmids, Enzymes, and Chemicals

Twelve Bacillus strains preserved in the laboratory, including 3 strains of *Bacillus subtilis*, 2 strains of *Bacillus atrophaeus*, 2 strains of *Bacillus sonorensis*, 3 strains of *Bacillus amyloliquefaciens*, *Bacillus aryabhattai*, and *Bacillus methylotrophicus*, were used for gene screening. The pMD-19 T vector (Takara, Dalian, China) was used for cloning and sequencing. *E. coli* DH5α competent cells (Tsingke, Beijing, China), *E. coli* BL21(DE3) cells (Vazyme, Nanjing, China), the pET28a(+) vector (Yuanye, Shanghai, China), and the pET-duet vector were used for protein overexpression. Restriction enzymes *Nde* I, *Not* I, *Mlu* I, *Sac* I, *Sal* I, *BamH* I, and *EcoR* I (Takara) and T4 DNA ligase (Vazyme) were used for plasmid manipulation and the ligation reaction, respectively. Substrates and standard chemicals were purchased from commercial sources, including 2,2’-azinobis(3-ehtylbenzothiazolin-6-sulfnic acid) ammonium salt (ABTS) (Aladdin, China); naphthalene, phenanthrene, anthracene, fluoranthene, pyrene, and benzo[a]pyrene (Macklin, China); and ampicillin, kanamycin, and isopropyl-beta-D-thiogalactopyranoside (IPTG) (Solarbio, Beijing, China).

### 2.2. Laccase Gene Screening

Several known *Bacillus* laccase sequences were obtained from NCBI, and multiple alignments were carried out with sequences showing high similarities using ClustalW (https://www.ebi.ac.uk/Tools/msa/clustalo/, accessed on 15 June 2021) and illustrated with ESPript 3.0 (http://espript.ibcp.fr/ESPript/ESPript/, accessed on 15 June 2021). Degenerate primers were designed according to conserved regions of several known laccase sequences. The forward B.SCotA/F and reverse B.SCotA/R primer sequences are shown in Table S1.

The genomic DNA of 12 *Bacillus* strains were used as DNA templates, and potential laccase genes were amplified by PCR. The PCR reaction system (50 μL) included 20 μL ddH_2_O, 25 μL 2× Taq Master Mix, 2 μL forward primer (10 μM), 2 μL reverse primer (10 μM), and 1 μL DNA template. The PCR procedure was 95 °C for 5 min; 35 cycles of 94 °C for 30 s, 50 °C for 30 s, and 72 °C for 1 min; and 72 °C for 10 min. The PCR product(s) was verified by 1.0% agarose gel electrophoresis, and the target band was excised using a gel DNA extraction kit (Vazyme). The PCR fragment and the pMD-19 T vector were ligated using the TA clone kit (Takara). Positive clones were confirmed by DNA sequencing by General Biosystems Co., Ltd. (Chuzhou, China).

### 2.3. Cloning and Overexpression of Lach5 in E. coli

Specific primers containing restriction sites were designed according to the gene sequence. Using *B. atrophaeus* YK5 genome DNA as the template, the target fragment was amplified using the forward primer LacH5-F/*Nde* I and reverse primer LacH5- R/*Not* I (sequences are shown in Table S1). The PCR reaction and gel excising system used were the same as described above. The PCR product and the pET28a expression vector were digested with *Nde* I and *Not* I for 5 h at 37 °C. Ligation of the PCR fragment and pET28a vector was carried out using T4 DNA ligase overnight at 16 °C, and the resulting recombinant plasmid, pET-LacH5, was transformed into *E. coli* BL21 (DE3) for heterologous expression. 

A positive clone was inoculated into a 250 mL flask containing 100 mL Luria–Bertani (LB) broth medium with 50 μg/mL kanamycin and cultured at 37 °C with constant shaking at 180 rpm. A total of 0.5 mM IPTG and 0.25 mM CuSO_4_ was added to induce gene expression when the optical density of the culture at 600 nm reached 0.6–0.8. After further incubation at 16 °C for 30 h, the cells were harvested by centrifugation (8000 *g*, 10 min, 4 °C), and the cells were resuspended in sodium phosphate buffer (50 mM NaH_2_PO_4_, 300 mM NaCl, pH 8.0). The cells were lysed by sonication, and the cell extract was centrifuged for 20 min at 12,000 *g* and 4 °C. The resulting supernatant was collected as a crude enzyme and purified using Ni-NTA affinity chromatography. The recombinant protein was eluted with washing buffer containing 250 mM imidazole. The concentration of purified recombinant protein was determined using a NanoDrop™ 1000 spectrophotometer (Thermo Scientific Co., Ltd., Waltham, MA, USA), and purity and molecular weight was examined by sodium dodecyl sulfate-polyacrylamide gel electrophoresis (SDS-PAGE).

### 2.4. Enzyme Assay of LacH5

Laccase activity was assayed with ABTS as the substrate. The reaction mixture consisted of 500 μL citrate–phosphate buffer (pH 5.0), 1 mM ABTS, and 1.5 μg purified enzyme. The reaction was incubated for 2 min at 60 °C. The oxidation of ABTS was measured at 420 nm using a UV-2012PC spectrophotometer (UNICO, Shanghai, China). The control was the reaction solution with no enzyme added unless otherwise specified. One unit of enzyme activity was defined as the amount of enzyme required to oxidize 1 μmol of substrate per min. All assays were conducted in triplicate.

### 2.5. Biochemical Characterization of LacH5

The activity of LacH5 was measured at 30–80 °C in citrate–phosphate buffer (pH 5.0) to determine the optimal temperature of LacH5 activity. The thermostability of LacH5 was tested by pre-incubating the enzyme at 40–80 °C, and residual activity was determined at 20 min intervals. Experiments were performed at pH values ranging from 3 to 11 to determine the optimal pH of LacH5 activity. The control groups with no enzyme added were measured simultaneously. The pH stability of LacH5 was evaluated by pre-incubating the enzyme at different pH values from 3 to 11 at 4 °C for 1 h and then measuring the residual activity under optimal conditions. 

The effect of organic solvents (10% (*v*/*v*) acetone, dimethyl sulfoxide (DMSO), methanol, acetonitrile, cyclohexane, isopropanol, and ethanol), metal ions (1 and 5 mM Fe^3+^, Al^3+^, Zn^2+^, Mg^2+^, Ba^2+^, Co^2+^, Mn^2+^, Cu^2+^, and Ca^2+^), and surfactants (0.5% (*v*/*v*) Tween-80, Triton-100, SDS, ethylene diamine tetraacetic acid (EDTA), and cetyl trimethyl ammonium bromide (CTAB)) on enzyme activity were investigated. Residual activity was determined in citrate–phosphate buffer (pH 5.0) with 1 mM ABTS as the substrate. The relative activity of the recombinant enzyme without the addition of metal ions or chemical agents was defined as 100%. 

### 2.6. Benzo[a]pyrene Degradation by LacH5

The BaP degradation reaction was carried out in a 1 mL system that included citrate–phosphate buffer (pH 5.0), purified recombinant enzyme, and BaP dissolved in acetonitrile and was added to the reaction system in a final concentration of 1 μg/mL. The whole system was incubated at 37 °C for 24 h. The effects of ABTS and copper ions on LacH5 activity were examined by adding 1 mM ABTS and 0.25 mM Cu^2+^ to the reactions. The control groups with no enzyme added were measured simultaneously. The reactions were terminated by adding more than 50% (*v*/*v*) acetonitrile, and the supernatant was taken and filtered for high-performance liquid chromatography (HPLC) analysis. HPLC was carried out on a Zorbax SB-C18 (4.6 × 150 mm, 5.0 μm) column at 30 °C, and the mobile phase was acetonitrile/H_2_O (88%:12%, *v*/*v*) with a flow rate of 0.8 mL/min, a fluorescence excitation wavelength of 384 nm, and an emission wavelength of 406 nm. For the product assay, samples were extracted with ethyl acetate followed by passage of nitrogen gas over the sample until dry. For HPLC analysis, dried samples were redissolved in acetonitrile and passed through a 0.22 μm organic filter membrane [28].

### 2.7. Fusion of Vitreoscilla Hemoglobin and LacH5

Two fusion laccases were constructed with *Vitreoscilla* hemoglobin as the fusion partner to enhance LacH5 activity. LacH5-F-*Sac* I/up, lacH5-R-*Sal* I/up, vgb-F-*Not* I/down, and vgb-R-*Nde* I/down were used to amplify the corresponding lacH5 and hemoglobin vgb genes, respectively. The resulting PCR fragments were digested and ligated into the pET-Duet vector to obtain the expression vector pET-duet-lacH5-vgb. Similarly, the specific primers vgb-F-*BamH* I/up, vgb-R-*EcoR* I/up, LacH5-F-*Sal* I/down, and LacH5-R-*Nde* I/down were used to amplify vgb and lacH5, respectively, to obtain expression vector pET-Duet-vgb-lacH5. The primers used are shown in Table S1. Unless otherwise specified, biochemical characterization of fusion protein Lach5-vgb and Vgb-LacH5 was analyzed as described above.

PAH degradation experiments were carried out in 1.5 mL reaction solutions that included citrate–phosphate buffer (pH 5.0), purified recombinant enzyme, and various PAHs in a final concentration of 1 μg/mL. Degradation efficiency was determined by HPLC, and the structures and detection wavelength of the PAHs used in this study are shown in Table 1. The dissolved oxygen was determined in fermentation broth with a portable dissolved oxygen meter.

### 2.8. Strong Promoter Screening and Replacement

The bi-directional-reporter vector pAD123-RFP1 containing a green fluorescent protein and a red fluorescent protein was constructed for promoter screening. Twelve samples of *Bacillus* species genome DNA were extracted using the Bacterial DNA Kit (OMEGA, Norcross, GA, USA) and digested with *Sau3A* I. The resulting 0.5–1.5 kb fragments were gel-purified and ligated into *BamH* I-digested pAD123-RFP1 and then transformed into *E. coli* BL21(DE3). Green and red colonies were observed visually on LB agar plates and were further analyzed by a microplate reader (Thermo Scientific Co., Ltd., Waltham, MA, USA). Identified positive clones were sequenced for promoter analysis.

Promoter activity was measured by taking the positive clones and incubating those clones overnight in LB (Luria–Bertani) medium with 100 μg/mL ampicillin. These cultures were transferred to 5 mL LB medium and shaken at 180 rpm for 6 h. Samples were diluted 1:20 (*v*/*v*) in LB medium to a final volume of 200 µL in 96-well microplates. The fluorescence values after 6 h incubation when cells reached the exponential growth phase were measured using a microplate reader with an excitation wavelength of 485 nm and an emission wavelength of 535 nm to examine protein expression. The background fluorescence and absorbance of the medium were determined from LB media without adding cells and were subtracted from the readings of the other wells. The activity of each promoter was reported as fluorescence/OD_600_. The pAD123-RFP1 vector harboring the T7 promoter was used as a positive control. The strongest identified promoter was selected to replace the T7 promoter in the original vector using the ClonExpress Ultra One Step Cloning Kit (Vazyme). The recombinant plasmids LacH5-wzJ1 and LacH5-vgb-wzJ1 were constructed, and the recombinant proteins were expressed, purified, and their activity measured. Each sample was tested with three biologically independent replicates.

## 3. Results

### 3.1. Laccase Screening and Heterologous Expression

Laccase genes of 12 *Bacillus* strains were PCR amplified using degenerate primers. Six of these strains gave a target band with a size of ~1.5 kb (Figure S1a), which were sequenced after TA cloning into *E. coli* DH5α. The resulting sequences were analyzed by NCBI Blast, and the open reading frames (ORF) were predicted by ORF Finder (https://www.ncbi.nlm.nih.gov/orffinder/, accessed on 27 June 2021). Five sequences, including three from *Bacillus amyloliquefaciens* and two from *Bacillus subtilis*, were reported previously [3,4,7], and one sequence from *B. atrophaeus* exhibited 82% similarity with the *B. subtilis* cotA gene (accession number: NP_388511.1) and was identified as a laccase, termed *lach5*. The full-length sequence of the gene was 1542 bp, encoding a protein 513 amino acids in length.

*lach5* was amplified using specific primers and ligated into pET28a(+), which contains a strong T7 promoter and overexpressed in *E. coli* BL21(DE3) to obtain a recombinant protein with a His_6_-tag at the C-terminus. SDS-PAGE analysis gave a strong band representing the recombinant protein, with a molecular weight of 61 kDa (Figure S1b). This observation correlated well with the predicted molecular weight.

### 3.2. Biochemical Characterization of LacH5

The optimal temperature for activity was determined using ABTS as the substrate and a temperature range of 30–80 °C (Figure S3a). The result showed that the activity of LacH5 increased continuously as the temperature increased from 30 to 60 °C and exhibited maximum activity at 60 °C, followed by a slight decrease from 60 to 80 °C. More than 80% activity was retained at 80 °C. Thermal stability experiments showed that LacH5 activity decreased slowly with increasing incubation time. More significant decreases in LacH5 activity were observed as the temperature increased from 60 to 80 °C. Nonetheless, LacH5 retained 60% of the original enzyme activity when incubated at 80 °C for 80 min (Figure S3b).

The activity of LacH5 was measured at pH 3.0–11.0 (Figure S3c). The results showed that LacH5 activity increased progressively between pH 3.0 and 5.0, with an optimum at pH 5.0. The activity decreased rapidly at higher pH values. The effect of pH on LacH5 stability revealed that LacH5 was relatively stable between pH 3.0–8.0 and exhibited a reduction in activity at pH < 3.0 and no activity at pH > 9.0 (Figure S3d).

### 3.3. Effect of Metal Ions, Surfactants, and Organic Solvents on the Activity of LacH5

The effects of metal ions on LacH5 activity were examined (Figure S4). The activity of LacH5 was inhibited strongly by the presence of Fe^3+^, Co^2+^, and Mn^2+^, but not affected after adding 1 mM or 5 mM Al^3+^, Zn^2+^, Mn^2+^, Ba^2+^, or Ca^2+^. Enzyme activity was enhanced by adding Cu^2+^ to almost 140% of the maximum activity, suggesting that Cu^2+^ assisted in activating the enzyme, and this observation is consistent with the previous reported laccase activity [29].

The effects of organic solvents and surfactants on LacH5 activity were also assessed (Table S2). LacH5 activity was inhibited strongly when acetone, SDS, Tween-80, Triton-100, acetonitrile, isopropanol, ethanol, and DMSO were present in the reaction. The metal chelating agent EDTA and CTAB also inhibited LacH5 activity. However, cyclohexane clearly enhanced the activity of the enzyme. Enhanced activity of LacH5 in the presence of Cu^2+^ and the significant inhibition effect by EDTA supports the existence of the copper ion active center in LacH5 and its vital role in enzyme activity [3].

### 3.4. Degradation of BaP by LacH5

**Figure 1:**
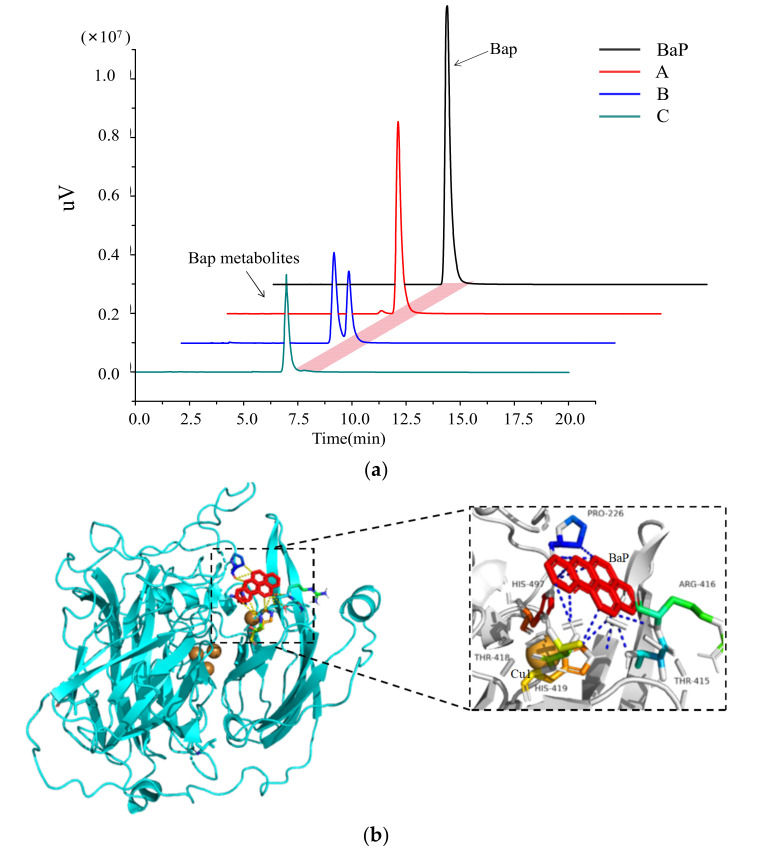
Analysis of BaP degradation and molecular docking results by LacH5. (**a**) HPLC analysis of the degradation of BaP by LacH5. The oxidation products of BaP were tested after incubating for 24 h. (A) only purified enzyme LacH5; (B) LacH5 and ABTS (1 mM); (C) LacH5, ABTS (1 mM), and CuSO4 (0.25 mM) were added in the BaP degradation reaction solution. (**b**) Visualization of the molecular docking results using PyMOL. The 3D structure prediction of LacH5 is shown in aquamarine, and the substrate BaP is shown in the red stick representation.

BaP oxidation by LacH5 was determined after incubating the reaction for 24 h. The degradation of BaP and the formation of oxidation products were analyzed by HPLC (Figure 1a). The results show that only 40% of BaP was oxidized by LacH5. The degradation efficiency increased to 72% in the presence of ABTS, which generally serves as a laccase mediator for enhancing enzyme oxidation. In contrast, BaP as high as 99% was oxidized after adding both copper ions and ABTS.

Molecular docking was carried out using Autodock vina, part of the Chimera software package, with LacH5 as the enzyme and BaP as the ligand. The docking results were visualized using PyMOL (Figure 1b). The conformation with the lowest free energy among the nine resulting conformations was selected for analyzing protein–ligand interactions. The docking results show that the substrate formed multiple intermolecular interactions with residues Pro226, Tyr415, Arg416, Tyr418, His419, and His497, which likely stabilized the substrate binding by forming hydrophobic interactions and van der Waals forces. Among these amino acids, His419 and His497 are coordinated with T1 Cu, which is responsible for electron transfer and substrate oxidation.

### 3.5. Construction and Expression of Fusion Proteins

One O_2_ molecule acquires four electrons and is reduced to two H_2_O molecules, according to the catalytic mechanism of laccases. Therefore, the amount of dissolved oxygen in the reaction plays a critical role in regulating the catalytic efficiency of laccases [16]. *Vitreoscilla* hemoglobin (VHb), which is encoded by the *vgb* gene, is an oxygen-binding protein that increases the oxygen supply, thereby enhancing cell growth, product synthesis, and stress tolerance, and has been used in the field of metabolic engineering for microorganisms, plants, and animals [30]. We hypothesized that the dissolved oxygen level should increase through the fusion of vgb and LacH5, leading to an increase in the catalytic efficiency of LacH5. 

Two fused expression plasmids, pET-Duet-lacH5-vgb (LacH5-vgb) and pET-Duet-vgb-lacH5 (Vgb-lacH5), were constructed (Figure S2a). The purified fusion proteins LacH5-vgb and Vgb-lacH5 were analyzed by SDS-PAGE. The observed presence of a clear target band with a molecular mass about 80 kDa in the SDS-PAGE gel is consistent with the predicted molecular weight (Figure S2b).

### 3.6. Characterization of Fusion Laccases

**Figure 2:**
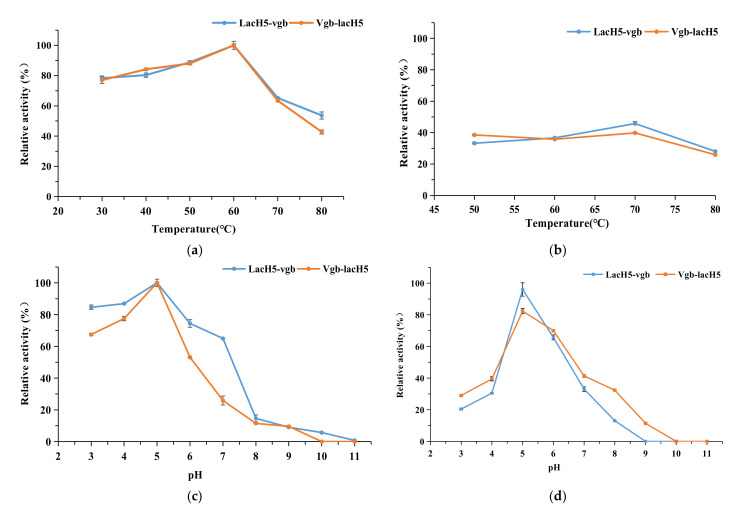
Enzymatic characterization of fusion proteins Lach5-vgb and Vgb-LacH5. (**a**) Effect of temperature on activity of Lach5-vgb and Vgb-LacH5; (**b**) effect of temperature on stability of Lach5-vgb and Vgb-LacH5; (**c**) effect of pH on activity of Lach5-vgb and Vgb-LacH5; (**d**) effect of pH on and stability of Lach5-vgb and Vgb-LacH5. (**a**–**d**) used ABTS as substrate. Note: The thermostability was studied in citrate–phosphate buffer (pH 5.0) by pre-incubating from 50 °C to 80 °C for 80 min.

Fusion proteins LacH5-vgb and Vgb-lacH5 showed strong activity in the temperature range of 30–60 °C, with the highest activity at 60 °C (Figure 2a), and a slight decline was observed when the temperature increased over 60 °C. The thermostability experiment indicated that 50% relative activity remained after 80 min at 50–70 °C, whereas only 25% residual activity was retained at 80 °C in the same incubation time, as shown in Figure 2b.

Increased activity of two fusion laccases was observed as pH from 3.0 to 5.0 and exhibited a maximum activity at pH 5.0, and gradually decreased when the pH was above 5.0 (Figure 2c). The relative activity of LacH5-vgb was as high as 86%, and was 56% and 10% higher than that of LacH5 and vgb-LacH5 at pH 3.0, respectively. The impact of pH on the stability of the fusion proteins was examined, as shown in Figure 2d. Two fusion laccases were stable at pH 5.0 for 1 h and retained more than 70% activity, indicating that they were more acid resistant than LacH5.

**Figure 3:**
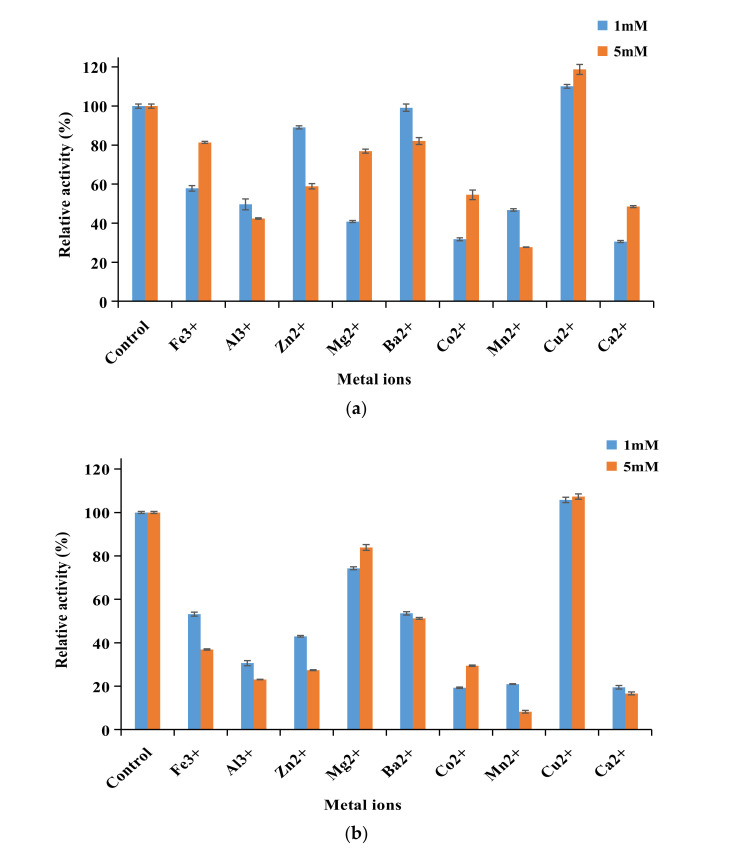

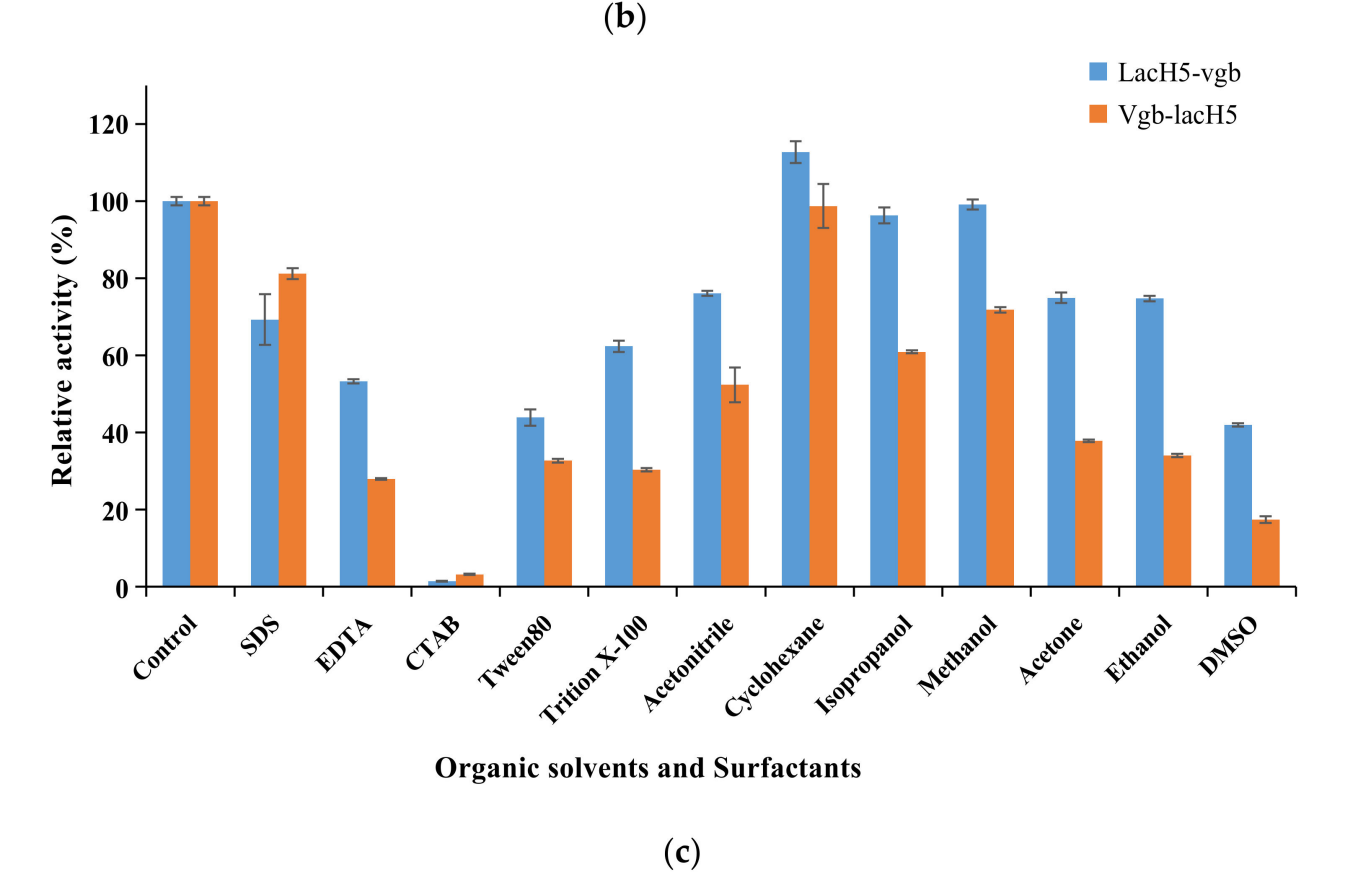
Effect of metal ions on activity of Lach5-vgb (**a**) and Vgb-LacH5 (**b**) and effect of surfactants and organic solvents on the activity of Lach5-vgb and Vgb-LacH5 (**c**). Residual activity was determined in citrate–phosphate buffer (pH 5.0) with 1 mM ABTS as substrate at 60 °C.

The effect of metal ions on the two fusion enzymes’ activity was studied. LacH5-vgb exhibited stronger tolerance to the tested metal ions compared to Vgb-lach5 (Figure 3a,b). The activity of the two fusion laccases was not affected by the addition of Mg^2+^, but was strongly inhibited by the presence of Fe^3+^, Al^3+^, Co^2+^, Mn^2+^, and Ca^2+^, and a similar inhibitory effect was also observed in the case of LacH5 (Figure S4). Significant fusion enzyme activity was enhanced by the addition of Cu^2+^ to 120 and 110% of the maximum activity, respectively, suggesting that Cu^2+^ assisted in increasing the two fusion laccases’ activity.

The organic solvent showed more obvious impact on the activity of vgb-LacH5 (Figure 3c). EDTA and Tween80 negatively affected the activity of the two fusion laccases. However, LacH5-vgb showed higher tolerance towards isopropanol and methanol, with an increase in relative activity by 2.0-fold and 2.6-fold than LacH5 and vgb-LacH5, respectively. The activity of two fusion laccases was improved by 10% in the presence of cyclohexane. It is interesting that the activity of LacH5 was strongly inhibited by SDS, but the activity of the fusion enzymes was less inhibited and still retained more than 60% residual activity.

Kinetic experiments indicated that the *Kcat*/*Km* values of LacH5-vgb and Vgb-lacH5 improved 1.83- and 1.55-fold when compared with that of the wild-type enzyme. Moreover, LacH5-vgb and Vgb-lacH5 showed a higher activity than LacH5 of 14.2 U/mg, which were 22.5 U/mg and 18.6 U/mg, respectively. These results coincided with an increase in the dissolved oxygen level in the fermentation broth of 65% and 46%, respectively (Table S3).

### 3.7. Fusion Protein Enhances the Degradation Efficiency of PAHs

**Figure 4:**
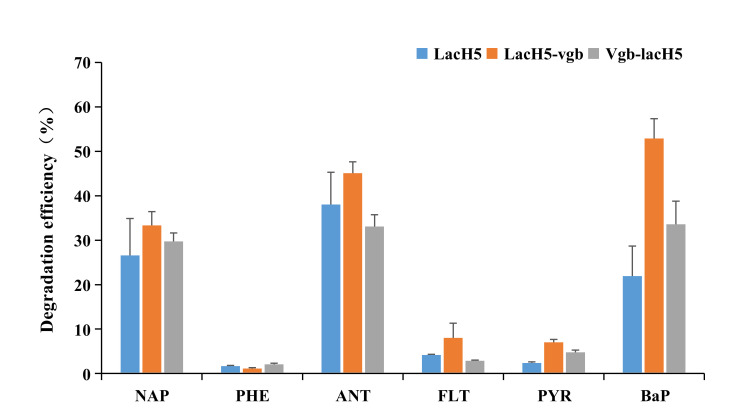
The degradation efficiency of PAHs by LacH5, LacH5-vgb, and Vgb-lacH5. Tested PAHs include NAP (naphthalene), PHE (phenanthrene), ANT (anthracene), FLT (fluoranthene), PYR (pyrene), and BaP (benzo[a]pyrene). The degradation efficiency of the PAHs was tested after incubating for 24 h. The standard deviations are expressed as mean bars.

**Figure 5:**
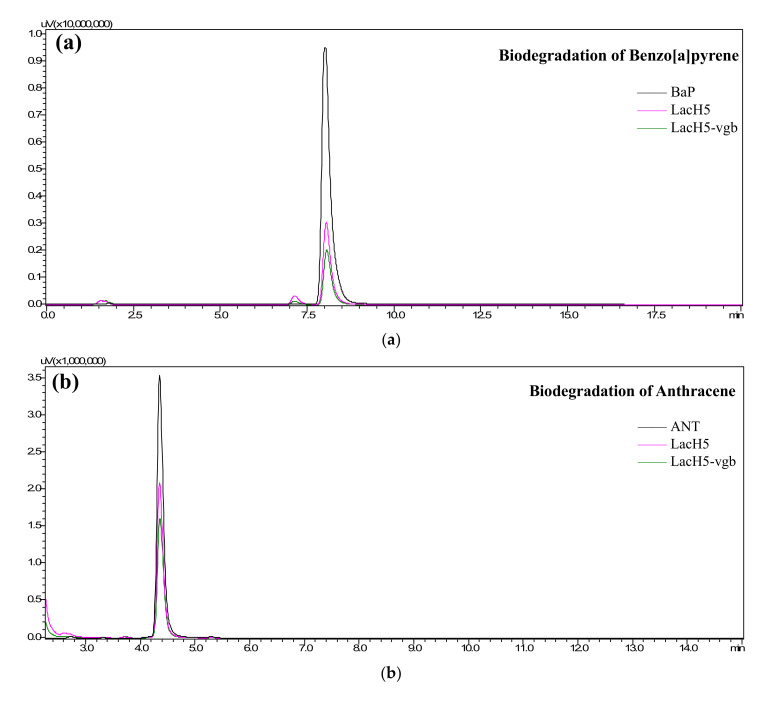

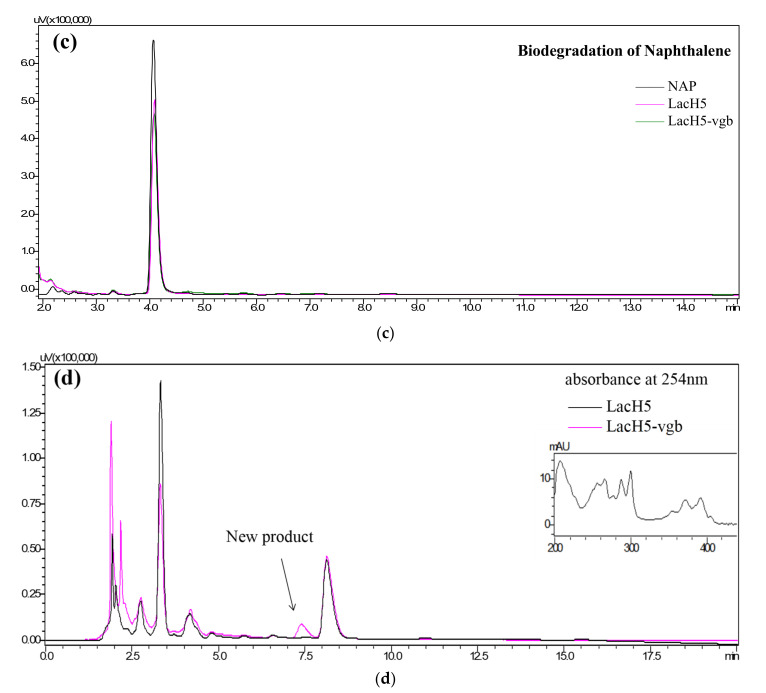
Biodegradation (37 °C, pH 5.0, 24 h) and control HPLC chromatograms of (**a**) benzo[a]pyrene, (**b**) anthracene, and (**c**) naphthalene. (**d**) HPLC analysis and comparison of the products of BaP degraded by LacH5 and LacH5-vgb. Note: The UV-visible spectrum of the new product is displayed as an inset.

The degradation efficiency of six PAHs was analyzed by HPLC after 24 h incubation (Figure 4). NAP, ANT, and BaP were effectively degraded by two fusion laccases (Figure 5a–c), but the remaining PAHs were resistant to enzymatic oxidation. The degradation efficiencies of NAP, ANT, and BaP by LacH5 were only 26.6%, 38.0%, and 21.9%, respectively. After fusion with VHb, a higher transformation rate towards NAP and ANT was achieved by LacH5-vgb, which was seen as an increase to 33.3% and 45.1%, respectively. In particular, the degradation efficiency of LacH5-vgb towards BaP was as high as 57.6%, resulting in 31% enhancement compared with the original enzyme LacH5. Vgb-lach5 also had a positive effect on the oxidation of the three PAHs, with only 12% improvement in BaP degradation, but was not as effective as LacH5-vgb.

Besides, a new methoxy derivative, 6-methoxy-benzo[a]pyrene, was unexpectedly detected by HPLC analysis in the process of measuring fusion protein degradation products. It was also characterized by a single absorption at 245 nm in the UV-visible spectrum and a molecular weight of 282.21 by GC-MS analysis (Figure 5d and Figure S5).

### 3.8. Strong Promoter Replacement Increases the Expression Level of LacH5

**Figure 6:**
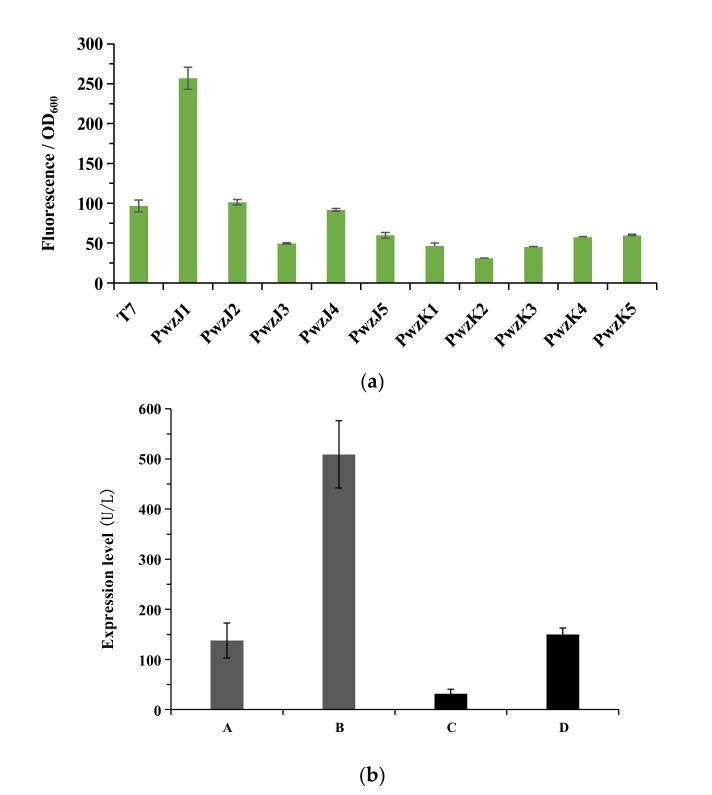
Promoter activity evaluation and the effect of strong promoter replacement. (**a**) Promoter activity evaluation. The activity of promoters was reported as fluorescence/OD600 after 6 h incubation. (**b**) The effect of a strong promoter on the expression levels of LacH5 and LacH5-vgb. (A) LacH5 with T7 promoter; (B) LacH5 with PwzJ1 promoter; (C) LacH5-vgb with T7 promoter; (D) LacH5-vgb with PwzJ1 promoter.

A bi-directional reporter plasmid pAD123-RFP1 with a red–green fluorescent protein as the reporter was constructed. Ten promoters were identified from 12 *Bacillus* species libraries through fluorescence/OD_600_ measurements (Figure 6a). PwzJ2 and PwzJ4 exhibited 105% and 95% fluorescence/OD_600_ when compared with that of the T7 promoter, indicating that their activity is comparable to that of the T7 promoter. PwzJ1 exhibited the highest fluorescence/OD_600_ which was approximately three times than that of the T7 promoter. Sequence analysis showed that PwzJ1 consisted of two promoters in tandem (Table S4). The other seven promoters showed lower fluorescence/OD_600_ values and were only approximately 30–60% of the T7 promoter.

Therefore, PwzJ1 was selected to replace the T7 promoter in the original vector to enhance recombinant enzyme expression levels. Two recombinant plasmids, LacH5-wzJ1 and LacH5-vgb-wzJ1, were constructed and transformed into *E. coli* BL21(DE3), followed by fermentation and purification. After incubation, the expression levels of LacH5-wzJ1 and LacH5-vgb-wzJ1 were 509.11 U/L and 149.87 U/L, respectively, which was 3.7-fold and 7.02-fold higher, respectively, than the wild-type enzyme (Figure 6b).

## 4. Discussion

Compared with fungal laccases, bacterial laccases have notable advantages, including high thermostability, a wide pH range of activity, and organic solvent tolerance. Thus, bacterial laccases are promising industrial catalysts. The well-characterized CotA-laccases are from the *Bacillus* genus, especially *Bacillus amyloliquefaciens* and *Bacillus subtilis* [7,25,29,31]. In the present study, a laccase gene, *lach5*, was identified from the genomic DNA of *B. atrophaeus* by sequence-based screening, which exhibited only 82% similarity with the *B. subtilis* cotA gene, and heterologous expression in *E. coli* was successful. Thermostability experiments showed that LacH5 retained more than 60% of its activity after incubating at 80 °C for 80 min, which is similar to the heat resistance X1 laccase from *B. subtilis* [31], but strikingly more heat resistant than the laccase from *B. licheniformis* DSM13, which lost approximately 92% activity after 1 h incubation at 80 °C [6]. This observation suggests that LacH5 has remarkable thermostability. The optimum pH for LacH5 oxidation of ABTS was 5.0, which is close to the optimal pH for CotA-laccases from other *Bacillus* species, such as the laccase from *B. pumilus* W3 with an optimum pH of 4.6 [32] and *B. licheniformis* laccase with an optimum pH of 4.2 [6].

Catalytic activity and the expression level are critical indicators to determine the suitability of laccases for industrial production. However, the activity and expression level of the native bacterial laccases are low when compared with those of fungal laccases, thus limiting their practical applications [1,2]. Many researchers have paid attention to bacterial laccase engineering. Several enzyme engineering techniques are available to address these issues, including heterologous expression and rational, semi-rational, or directed evolution [26,33]. *E. coli*, *B. subtilis*, and *P. pastoris* are used widely as heterologous expression hosts. *E. coli* is easy to genetically manipulate for CotA laccase expression compared to *B. subtilis* and *P. pastoris* [34]. In addition, genetic manipulation, functional complementation cloning, and high-frequency DNA transformation of *E. coli* are easy. Although random mutation can effectively improve catalytic activity and thermal stability of target enzymes, it takes considerable time and effort to identify an enhanced laccase mutant because of the absence of simple phenotype-screening methods [35]. A better method needs to be explored to improve the catalytic efficiency and expression level.

Laccase catalysis involves three steps [2,15]. Initially, T1 Cu is reduced and gains four electrons donated by a reducing substrate. Then, internal electron transferring from T1 Cu to T3 Cu and T2 Cu through a Cys–His pathway occurs that is highly conserved among multicopper oxidases. Finally, oxygen is reduced to water at the T2/T3 trinuclear cluster center [36]. Oxygen plays a key role as an electron acceptor in the laccase catalysis process, and the amount of dissolved oxygen in the reaction solution has a significant effect on substrate oxidation. We hypothesized that high dissolved oxygen levels should provide more electron acceptors, thus accelerating the reaction rate and improving the catalytic efficiency of LacH5. VHb was the first soluble heme-binding protein discovered from a bacteria with a fast rate of oxygen dissociation and is used widely in various biological research fields, such as biochemical engineering, metabolic engineering, and bioremediation [30,37]. In this study, two Vgb-based LacH5 fusion constructs were prepared to increase the amount of dissolved oxygen and thus enhance the catalytic efficiency of LacH5. The results indicate that the catalytic efficiency of the two fusion laccases Vgb-lacH5 and LacH5-vgb was increased by 1.55- and 1.83-fold, respectively. The dissolved oxygen in the two fermentation broths was 5.11 mg/mL and 5.76 mg/mL, respectively, which was much higher than the LacH5 fermentation broth (3.5 mg/mL), showing that the increase in dissolved oxygen enhanced the catalytic efficiency of LacH5 significantly.

Previous research has shown that the oxidation process of ABTS by laccase can be divided into two steps [38]. ABTS is initially oxidized to form the ABTS^+^^•^ cation radical, which is followed by oxidization to the ABTS^2+^ cation. The first step is faster than the second step. Thus, the second step is the rate-determining step, which is facilitated by an oxygen molecule. Complete oxidation of ABTS to ABTS^2+^ leads to decolorization of the solution [38]. The oxidation experiments with ABTS revealed that LacH5-vgb decolorized ABTS entirely in 10 h, whereas LacH5 did not lead to obvious decoloration even after 24 h (Figure S6), suggesting that the increase in the amount of dissolved oxygen promoted the oxidation of ABTS notably. This observation further confirmed that the amount of dissolved oxygen in the fermentation broth is a pivotal factor in improving the catalytic efficiency of the laccase.

It has been demonstrated that laccase can degrade a variety of PAHs effectively [28,39,40]. CotA laccase is one of the most characterized laccases from *Bacillus subtilis*. Jun Zeng et al. studied the transformation of CotA and *E. coli* CueO laccase on 15 PAHs and found that partially purified CotA and CueO only oxidized two of the 15 tested PAHs, i.e., ANT and BaP [41]. Besides, the degradation activity of CotA on PAHs was stronger than that of CueO. In the present study, the bacterial laccase LacH5 not only degraded ANT and BaP effectively, but also exhibited significant degrading activity toward NAP. These results indicate that laccases are not able to degrade all kinds of PAHs, and that only a fraction of PAHs can be degraded. It should be noted that laccase CotA from *B. subtilis* almost completely degraded 0.5 ug BaP after 24 h co-incubation with ABTS and Cu^2+^ [41], whereas LacH5 could completely degrade 1 ug BaP under the same conditions, indicating that the degradation efficiency of BaP by LacH5 was significantly higher than that of CotA. Moreover, the fusion protein LacH5-vgb could degrade BaP up to 1.31 ug.

Copper ions can enhance laccase gene expression at the genetic transcription level and incorporate with active sites of enzyme during laccase synthesis, thus stimulating laccase activity [42,43]. Shi et al. reported that laccase activity was enhanced by 0.4–1 mM of Cu^2+^ [44]; similarly, the activity of laccase from *Streptomyces griseorubens* JSD-1 was increased by 28% after adding 1 mM Cu^2+^ to the reaction solution [45]. In our experiments, the activity of both LacH5 and the fusion protein were improved after the addition of copper ions, suggesting that the activity of recombinant laccases was copper dependence.

Previous studies revealed that oxidation mediators, like ABTS, which play a critical role in electron transfer and dioxygen reduction, effectively enhance the activity of laccase [28,46,47]. Zeng et al. reported that the oxidation efficiency of CotA against BaP was significantly increased after the addition of ABTS [41]. Moreover, laccase from *Pleurotus ostreatus* D1 was able to degrade PAHs only in the presence of ABTS or another synthetic mediator [48]. The laccase LacH5 can effectively degrade three PAHs with the addition of ABTS. The degradation activity of BaP especially was increased by 32%, which is consistent with the degradation of PAHs by CotA laccase. In contrast, the degree of PAH oxidation by the fungal laccase from *T. versicolor* was significantly decreased in the presence of ABTS [28].

Among the six PAHs tested, LacH5 was able to degrade BaP, ANT, and NAP notably, and the degradation efficiency was further improved by laccase fusion with VHb. It has been reported that the oxidation of PAHs by laccase is closely related to its ionization potentials (IPs) [49]. PAHs with lower IP values are easily oxidized; therefore, LacH5 was able to oxidize ANT (7.43 eV) and BaP (7.06 eV), but was inactive with PHE (8.19 eV) and FLT (7.95 eV). Paradoxically, LacH5 could oxidize NAP (8.13 eV) with a high IP value, and a similar result was also observed in the degradation of PHE (8.19 eV) by laccase from *L. gongylophorus*. A possible explanation to this effect is the bulk and steric hindrance of different PAHs in space [50]. NAP and PHE with a smaller size only contain two or three aromatic rings, making them easier to enter the active center of laccase and be oxidized [48]. In summary, the degradation efficiency of PAHs by laccase may be affected by the addition of ABTS and copper ions, ionization potentials, and molecular structure of PAHs.

In addition, a new chromatographic peak was detected in the LacH5-vgb reaction solution by HPLC analysis. The corresponding molecular weight of the product was determined to be 282.21 by GC-MS analysis (Figure S5), and the UV absorption spectrum was consistent with the spectrum of 6-methoxy-benzo[a]pyrene reported previously. Therefore, the product was identified as 6-methoxy-benzo[a]pyrene. Zeng et al. [51] reported that BaP was oxidized and hydroxylated as the intermediate 6-hydroxy-benzo[a]pyrene, which can be further oxidized to benzo[a]pyrene 1,6-, 3,6- and 6,12-quinones primarily by laccase from *Trametes versicolor*. The intermediate can also be converted into methoxy-benzo[a]pyrene by catechol-o-methyltransferase from pyrene-degrading mycobacteria. In this study, LacH5-vgb degraded BaP to 6-methoxy-benzo[a]pyrene directly, indicating that the fusion expression of LacH5 and VHb increased the dissolved oxygen and resulted in the new product and provided direct evidence for BaP degradation.

Strong promoter replacement is an effective method to increase enzyme yield and has been applied widely in enzyme engineering and synthetic biology [52]. For example, a new promoter, pShuttle-09, which showed eight times higher activity than the strong promoter P43, was characterized from *B. licheniformis* genomic DNA. Replacing the original promoter with pShuttle-09 improved the β-Gal production level by 60% [53]. In the present study, 10 new promoters were screened from 12 strains of *Bacillus* genomic DNA libraries using a bi-directional promoter-screening plasmid. Among them, the novel promoter PwzJ1 exhibited the strongest fluorescence intensity in the activity assay, and this activity was almost three times stronger than that of the T7 promoter. Replacing the T7 promoter with PwzJ1 increased the expression levels of the recombinant enzyme LacH5 and the fusion protein LacH5-vgb by 3.7-fold and 7.0-fold, respectively, and further confirmed that promoter engineering is an effective procedure for increasing enzyme expression levels.

## 5. Conclusions

A laccase LacH5 with remarkable thermostability was characterized from *B. atrophaeus*. LacH5 fused with the vgb gene increased the amount of dissolved oxygen and enhanced the laccase catalytic efficiency significantly, and clearly improved the oxidative rate of ABTS. In particular, the LacH5-vgb fusion protein exhibited stronger degradation efficiency toward three PAHs of six. In particular, a new product, 6-methoxy-benzo[a]pyrene, was identified when BaP was oxidized. Notably, recombinant enzyme expression was increased significantly by replacing the T7 promoter with the strong promoter PwzJ1, which was screened from B. sterols. The current study provides a new candidate laccase for PAH degradation and develops a novel engineering approach to improve the activity and expression level of recombinant laccases.

## Figures and Tables

**Table 1:** Main parameters of PAHs used in this study.

PAHs	Abbreviation	Structure	Rings	Excitation Wavelength	Emission Wavelength
Naphthalene	NAP	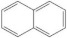	2	270	324
Phenanthrene	PHE	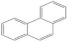	3	248	375
Anthracene	ANT	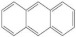	3	248	375
Fluoranthene	FLT	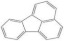	4	280	462
Pyrene	PYR	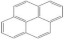	4	270	385
Benzo[a]pyrene	BaP	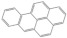	5	384	406

## Data Availability

Not applicable.

## Supplementary Materials

The following supporting information can be downloaded at: https://www.mdpi.com/article/10.3390/biology11081129/s1, Figure S1: (a) PCR amplified fragment of laccase gene from *Bacillus* using degenerate primers Lane M, DL2000 marker. Lane 1,6,7,8,11,12, Target band (b) SDS-PAGE analysis of LacH5. Lane M, standard protein marker. Lane 1, crude proteins of *E. coli* BL21 harboring plasmid pET28a-lacH5 with addition of 0.5 mM IPTG. Lane 2, eluted LacH5-1 with 250mM imidazole; Figure S2: (a) PCR amplified fragments of fusion protein Lane M, DL2000 marker. Lane 1, fusion fragment of LacH5-vgb Lane 2, fusion fragment of vgb-LacH5. (b) SDS-PAGE analysis of fusion protein Lane M, standard protein marker. Lane 1, eluted LacH5-vgb Lane 2, eluted Vgb-lacH5; Figure S3: Effect of temperature and pH on the activity and stability of LacH5. (a) Effect of temperature on activity. (b) Effect of temperature on stability. (c) Effect of pH on activity. (d) Effect of pH on stability; Figure S4: Effect of metal ions on LacH5 activity. Residual activity was determined in citrate-phosphate buffer (pH 5.0) with 1 mM ABTS as the substrate at 60 °C; Figure S5: Electron ionization mass spectrum of the new product from LacH5-vgb; Figure S6: Oxidation effect of LacH5 and the fusion protein toward ABTS. (a) Initial color status at room temperature. (b) Color status after incubation at room temperature for 24 h. 1, LacH5; 2, Vgb-lacH5; 3, LacH5-vgb. Table S1: Primers used in present study; Table S2: Effect of surfactants and organic solvents on the activity of LacH5; Table S3: Comparison of characteristics between LacH5 and the fusion protein; Table S4: Promoter PwzJ1 sequence (5′-3′).
