# Supplementary material for: Improving Degradation of Polycyclic Aromatic Hydrocarbons by Bacillus atrophaeus Laccase Fused with Vitreoscilla Hemoglobin and a Novel Strong Promoter Replacement
Source: Biology (Basel). 2022 Jul 27;11(8):1129. doi: 10.3390/biology11081129 (PMC9404780; doi:10.3390/biology11081129)
Supplement: Supplementary file 1 [file biology-11-01129-s001.zip › biology-1828296-supplementary.pdf]

Appendix A. Supplementary Materials

Improving degradation of polycyclic aromatic hydrocarbons by *Bacillus atrophaeus* laccase fused with *Vitreoscilla* hemoglobin and a novel strong promoter replacement

Luyao Wang, Yuzhi Tan, Shengwei Sun, Liangjie Zhou, Guojun Wu, Yuting Shao, Mengxi Wang, Zhihong Xin*

Affiliation: Key Laboratory of Food Processing and Quality Control, College of Food Science and Technology, Nanjing Agricultural University, Nanjing 210095, PR China

* Corresponding author

Postal address: College of Food Science and Technology, Nanjing Agricultural University, Nanjing, 210095 PR, China. E-mail: **xzhfood@njau.edu.cn** Tel./fax: +86 25 8439 5618

Table S1. Primers used in present study*

Primer	Nucleotide sequence (5′-3′)
B.SCotA/F	ATGRMMCTDGAAAARTTYGYHG
B.SCotA/R	TCM AKHGGHCKCATCATRTCRTARTC
LacH5-F/Nde I	GGAATTCCATATGAATC TTGAAAAATTTGCGG
LacH5- R/Not I	ATAAGAATGCGGCCGCTTGATTTGGATCCACAAC ATC
lacH5-F-SacI/up	GCGCGAGCTCATGAATCTTGAAAAATTTGCGG
lacH5-R-SalI/up	ACGCGTCGACTTGATTTGGATCCACAACATC
vgb-F-NotI/down	ATAAGAATGCGGCCGCTATGCTGGATCAGCAGA
vgb-R-NdeI/down	GGAATTCCATATGGTGGTGGTGGTGGTGGTGC
vgb-F-BamHI/up	CGCGGATCCATGCTGGATCAGCAGA
vgb-R-EcoRI/up	CCGGAATTCGTGGTGGTGGTGGTGGTGC
lacH5-F-SalI/down	ACGCGTCGACGTATGAATCTTGAAAAATTTGC
lacH5-R-NdeI/down	GAATTCCATATGTCAGTGGTGGTGGTGGTGGTGCT

*The underlined base is the restriction enzyme recognition site.

(a)

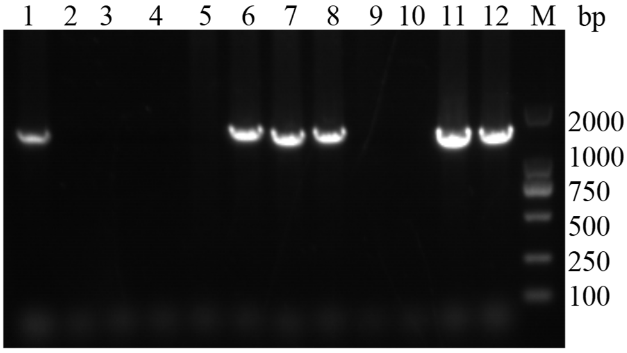

(b)

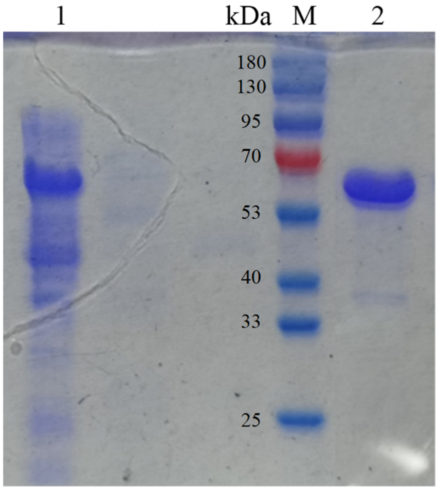

Figure S1. (a) PCR amplified fragment of laccase gene from *Bacillus* using degenerate primers

Lane M, DL2000 marker. Lane 1,6,7,8,11,12, Target band (b) SDS-PAGE analysis of LacH5.

Lane M, standard protein marker. Lane 1, crude proteins of *E. coli* BL21 harboring plasmid pET28a-lacH5 with addition of 0.5 mM IPTG. Lane 2, eluted LacH5-1 with 250mM imidazole.

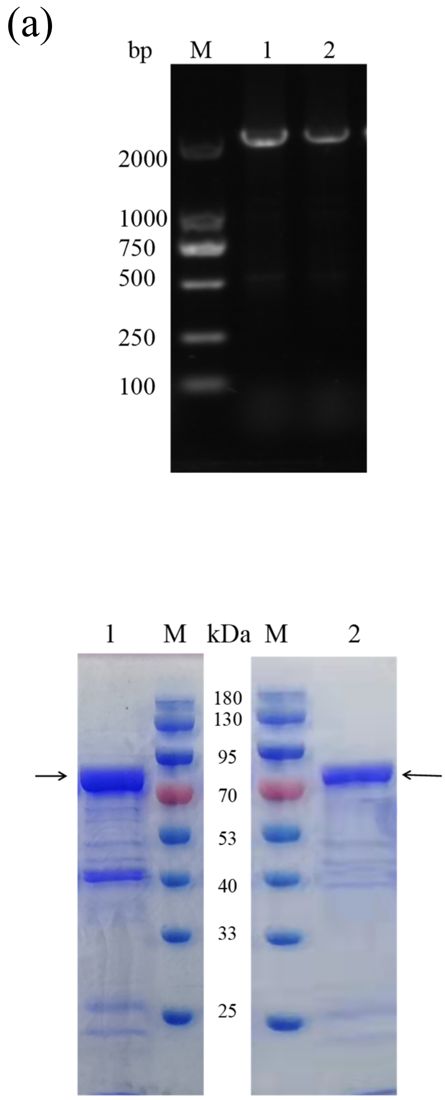

Figure S2. (a) PCR amplified fragments of fusion protein Lane M, DL2000 marker. Lane 1, fusion fragment of LacH5-vgb Lane 2, fusion fragment of vgb-LacH5. (b)SDS-PAGE analysis of fusion protein Lane M, standard protein marker. Lane 1, eluted LacH5-vgb Lane 2, eluted Vgb-lacH5.

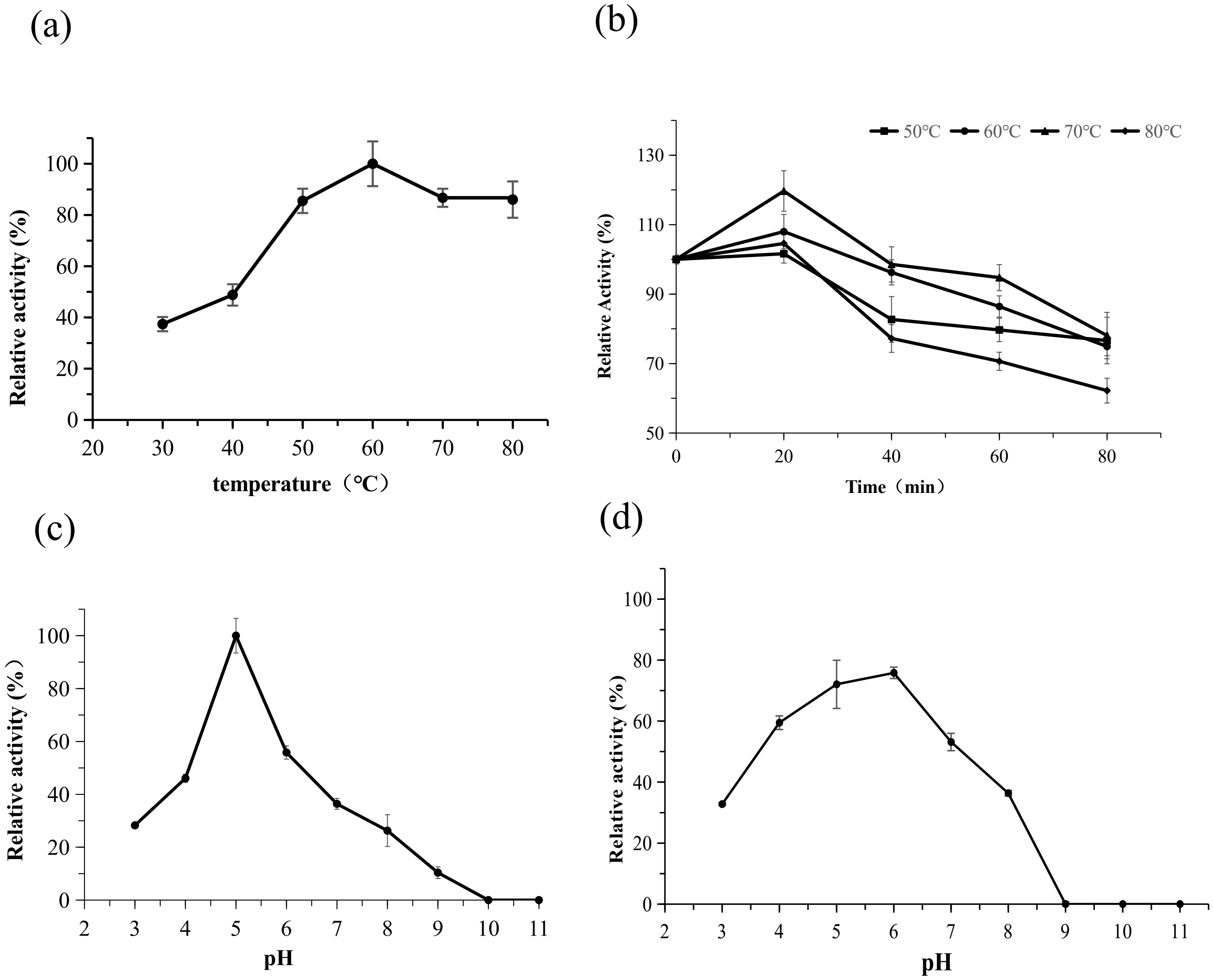

Figure S3. Effect of temperature and pH on the activity and stability of LacH5. (a) Effect of temperature on activity. (b) Effect of temperature on stability. (c) Effect of pH on activity. (d) Effect of pH on stability.

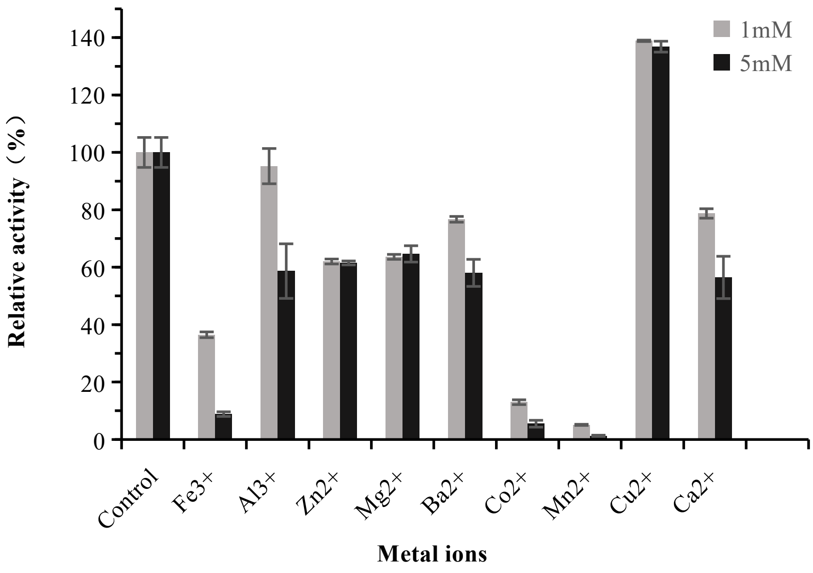

Figure S4. Effect of metal ions on LacH5 activity. Residual activity was determined in citrate-phosphate buffer (pH 5.0) with 1 mM ABTS as the substrate at 60 °C. The standard deviation were expressed as means bars.

Table S2. Effect of surfactants and organic solvents on the activity of LacH5*.

Chemicals	Concentration	Relative activity (%)
SDS	0.5% (w/v)	16.09 ± 0.5
EDTA	0.5% (w/v)	ND[a]
CTAB	0.5% (w/v)	ND
Tween80	0.5% (v/v)	63.13 ± 0.5
Trition X-100	0.5% (v/v)	35.03 ± 0.4
Acetonitrile	10% (v/v)	39.20 ± 0.8
Cyclohexane	10% (v/v)	117.88 ± 0.7
Isopropanol	10% (v/v)	46.85 ± 2.1
Methanol	10% (v/v)	38.03 ± 4.9
Acetone	10% (v/v)	58.28 ± 0.8
Ethanol	10% (v/v)	10.66 ± 1.4
DMSO	10% (v/v)	42.88 ± 3.8

*All assays were performed in triplicate, and the values are expressed as the mean ± standard deviation.

[a]ND: not detectable.

Table S3. Comparison of characteristics between LacH5 and the fusion protein*.

Enzyme	*K*m (μM)	*K*cat (S^{-1})	*K*cat/*K*m ($s^{-1}mM^{-1}$)	Catalytic efficiency	Dissolved oxygen (mg/ml)
LacH5	668.10 ± 62.84	218.26 ± 18.99	309.80 ± 22.96	1	3.51 ± 0.55
LacH5-vgb	336.58 ± 86.8	184.27 ± 48.24	568.21 ± 24.80	1.83	5.76 ± 0.52
Vgb-lacH5	263.95 ± 27.5	126.81 ± 9.50	481.58 ± 18.90	1.55	5.11 ± 0.59

*Laccase activity was determined using ABTS as the substrate (pH = 5.0, 60 °C).

Table S4. Promoter PwzJ1 sequence (5′-3′).

GATCAGCTCCTCGCCCTTGCTCACCATGTGCCACCTGACGTCTAAGAAACCATTAT<br>TATCATGACATTAACCTATAAAAATAGGCGTATCACGAGGCCCTTTCGTCTTCAAG<br>AATTCGAGCTCGGTACCCGGGGATCGGGCTTGCAGTACATGCATAATAAGAGGAA<br>AAGCAGGCGAGTGGTTATAAGCGCCTGCTTTTTTTTGTTGAAAGCGCTTTATTTTTC<br>CCCTACAATAGATGAAAACGGCGTGTAAGGGAGGAGCGATC

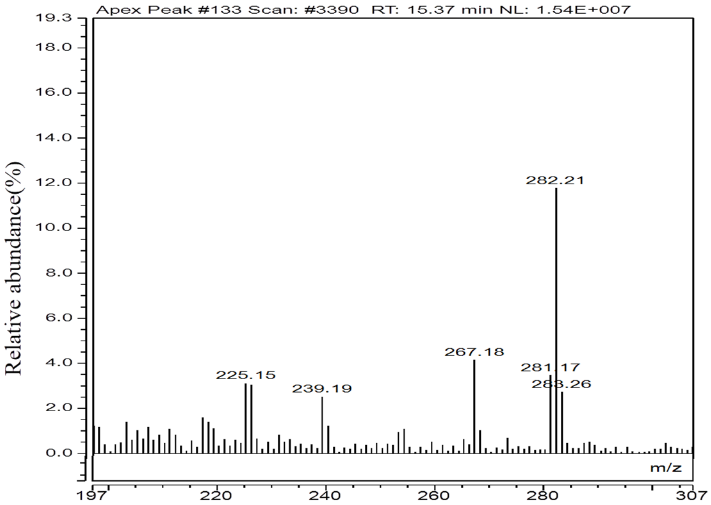

Figure S5. Electron ionization mass spectrum of the new product from LacH5-vgb.

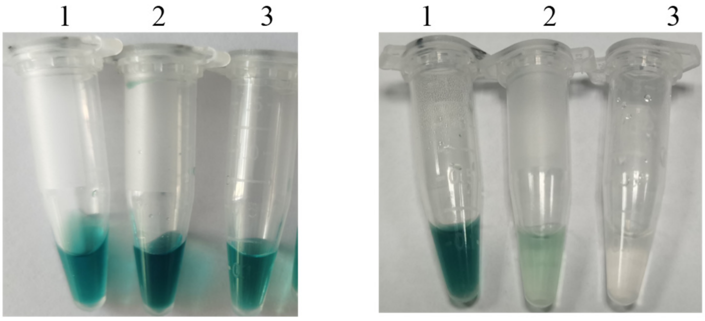

(a) (b)

Figure S6. Oxidation effect of LacH5 and the fusion protein toward ABTS. (a) Initial color status at room temperature. (b) Color status after incubation at room temperature for 24 h. 1, LacH5; 2, Vgb-lacH5; 3, LacH5-vgb.
